# Electrochemically Induced Interphase by Complex Hydride Anions in Argyrodite Solid Electrolytes for Stable Lithium Metal All‐Solid‐State Batteries

**DOI:** 10.1002/advs.75514

**Published:** 2026-05-09

**Authors:** Sangho Lee, Hyunseo Park, Ye‐Eun Park, Taehyun Kim, Taegyoung Lee, Taeseung Kim, SeoungJae Kang, Yerim Chae, Seunghee Joo, Jinkwang Hwang, Kyungsu Kim, Kazuhiko Matsumoto, Woosuk Cho, Sangryun Kim

**Affiliations:** ^1^ Department of Chemistry Gwangju Institute of Science and Technology (GIST) Gwangju Republic of Korea; ^2^ Advanced Batteries Research Center Korea Electronics Technology Institute (KETI) Seongnam Republic of Korea; ^3^ Graduate School of Energy Science Kyoto University Kyoto Japan; ^4^ Graduate School of Energy Convergence Gwangju Institute of Science and Technology (GIST) Gwangju Republic of Korea

**Keywords:** argyrodite, complex anions, Li metal battery, solid electrolyte

## Abstract

Complex hydride anion substitution in the argyrodite solid electrolyte has emerged as a promising approach to enhance ionic conductivity and interfacial stability. Despite these advances, the influence of complex hydride anions on Li metal interfacial stability remains unclear. Here, we clarify that complex hydride anions drive distinct interfacial reaction pathways at Li metal under electrochemical operation. In the BH_4_^−^‐substituted argyrodite Li_5_PS_4_(BH_4_)_2_, BH_4_^−^ species rapidly react with Li during electrochemical operation, forming a Li─B─H‐rich interphase. This interphase limits further decomposition of the sulfide framework while maintaining efficient Li^+^ transport. In contrast, the conventional halide argyrodite Li_6_PS_5_Cl undergoes sustained interfacial decomposition into Li_2_S and Li_3_P, resulting in unstable cycling behavior. Based on these results, we developed all‐solid‐state Li metal batteries with a gradual current increase that promotes Li─B─H‐rich interphase formation, achieving stable cycling over 1000 cycles at high current densities up to 2.1 mA cm^−2^. Collectively, our findings provide new insight into how complex hydride anions in solid electrolytes, when coupled with rationally engineered electrochemical operation, enable stable, high‐current all‐solid‐state Li metal batteries.

## Graphical abstract

Complex hydride anion substitution in argyrodite electrolytes has attracted attention for improving interfacial stability, yet the influence of these anions at Li metal interfaces remains unclear. This work shows that electrochemically formed BH_4_^−^‐derived interphases enable stable high‐current full‐cell cycling. Future research on electrochemically formed interphases involving complex hydride anions could significantly enhance performance and advance all‐solid‐state Li metal batteries.

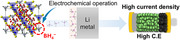

## 1 Introduction

As the demand for safer and higher energy‐density batteries continues to grow, Lithium (Li) metal is considered one of the most promising anode materials due to its extremely high theoretical capacity of 3860 mAh g^−1^ and the lowest electrochemical potential of −3.04 V *vs*. the Standard Hydrogen Electrode (SHE) [1, 2, 3]. However, in conventional Li‐ion batteries with liquid electrolytes, unstable Li deposition can form dendrites and cause internal short circuits [1, 4]. In this context, all‐solid‐state Li metal batteries using inorganic solid electrolytes have gained attention for improved safety and higher energy density [5]. To fully exploit the advantages of Li metal anodes in such systems, solid electrolytes should provide high Li ion conductivity and interfacial compatibility with Li metal.

Sulfide‐based solid electrolytes are promising candidates for all‐solid‐state Li metal batteries because of their high ionic conductivity and mechanical softness [6, 7, 8]. Despite these advantages, sulfide solid electrolytes exhibit a narrow electrochemical stability window (typically 1.7–2.3 V *vs*. Li/Li^+^) and are thermodynamically unstable against Li metal, forming interfacial decomposition products, including Li_2_S and Li_3_P [9, 10]. These decomposition products yield a mixed‐conductive, heterogeneous interphase that promotes ongoing side reactions during long‐term cycling [10, 11]. Therefore, a fundamental understanding of interfacial reactions is essential for achieving stable and long‐term operation of Li metal cells.

Complex hydrides, which generally follow the formula *M_x_* (*M*′*_y_* H*_z_*), where *M* denotes alkali cations and *M*′*_y_* H*_z_* corresponds to complex anions, have attracted considerable attention as promising solid electrolytes for all‐solid‐state Li metal batteries because of their high Li ion conductivity and excellent compatibility with Li metal [12, 13, 14, 15]. Specifically, owing to their hydrogen rich nature, complex hydride anions exhibit a strongly reducing character and are stable at low potentials, which has been exploited to enhance stability against metal electrodes [16, 17, 18]. In parallel, sulfide argyrodites such as Li_6_PS_5_ *X* (*X* = Cl^−^, Br^−^, and I^−^) provide high ionic conductivity, yet react with Li metal [19, 20, 21]. These considerations motivate incorporating complex hydride anions into the argyrodite framework by substituting BH_4_^−^ for halide anions at the *X* (Cl^−^, Br^−^, and I^−^) site [22, 23, 24].

In this context, incorporating BH_4_^−^ has been reported to improve ionic conductivity and electrochemical stability, leading to better cell performance [25, 26, 27, 28, 29, 30, 31]. To understand and rationally design such improvements, elucidating BH_4_^−^‐mediated interfacial reactions at the Li metal interface is essential. Previous work on physically mixed LiBH_4_‐Li_6_PS_5_Cl composites has provided useful insights into the interfacial behavior of BH_4_^−^ against Li metal [32]. However, when BH_4_^−^ is incorporated into the argyrodite lattice, its specific role at the Li metal interface remains insufficiently resolved. In particular, during Li metal cell operation, interfacial reactions can proceed through both chemical and electrochemical pathways, making it important to clarify their coupled contributions for reliable interpretation.

In this study, we identify complex hydride anions as the key factor that redirects the Li metal interfacial reaction pathway under electrochemical operation. By examining the hydride‐substituted argyrodite Li_5_PS_4_(BH_4_)_2_ and the halide argyrodite Li_6_PS_5_Cl, we show that chemically driven and electrochemically driven interfacial reactions in argyrodite electrolytes diverge markedly. Under purely chemical reaction conditions, both electrolytes form Li_2_S and Li*_x_* P at the Li/electrolyte interface. However, with electrochemical operation, interfacial phase evolution diverges. Li_6_PS_5_Cl undergoes electrochemical reduction along the same side‐reaction pathway established by chemical reaction. In contrast, for Li_5_PS_4_(BH_4_)_2_, chemically driven decomposition occurs at the Li interface, but once electrochemical operation begins, BH_4_^−^ species preferentially react with Li and direct the interfacial reactions toward BH_4_^−^‐derived products, leading to the formation of a Li─B─H‐rich interphase. This interphase suppresses further reduction of the PS_4_^3−^ framework while maintaining efficient Li^+^ transport, thereby enabling stable long‐term cycling in Li metal full cells. Furthermore, we find that the formation and stability of this interphase are highly sensitive to how the current is applied. Therefore, we propose that beyond hydride anion substitution in the argyrodite framework, establishing the interphase via electrochemical reaction is important for achieving reliable Li metal all‐solid‐state batteries.

## 2 Results and Discussion

### 2.1 Solid Electrolyte Characterization

**FIGURE 1:**
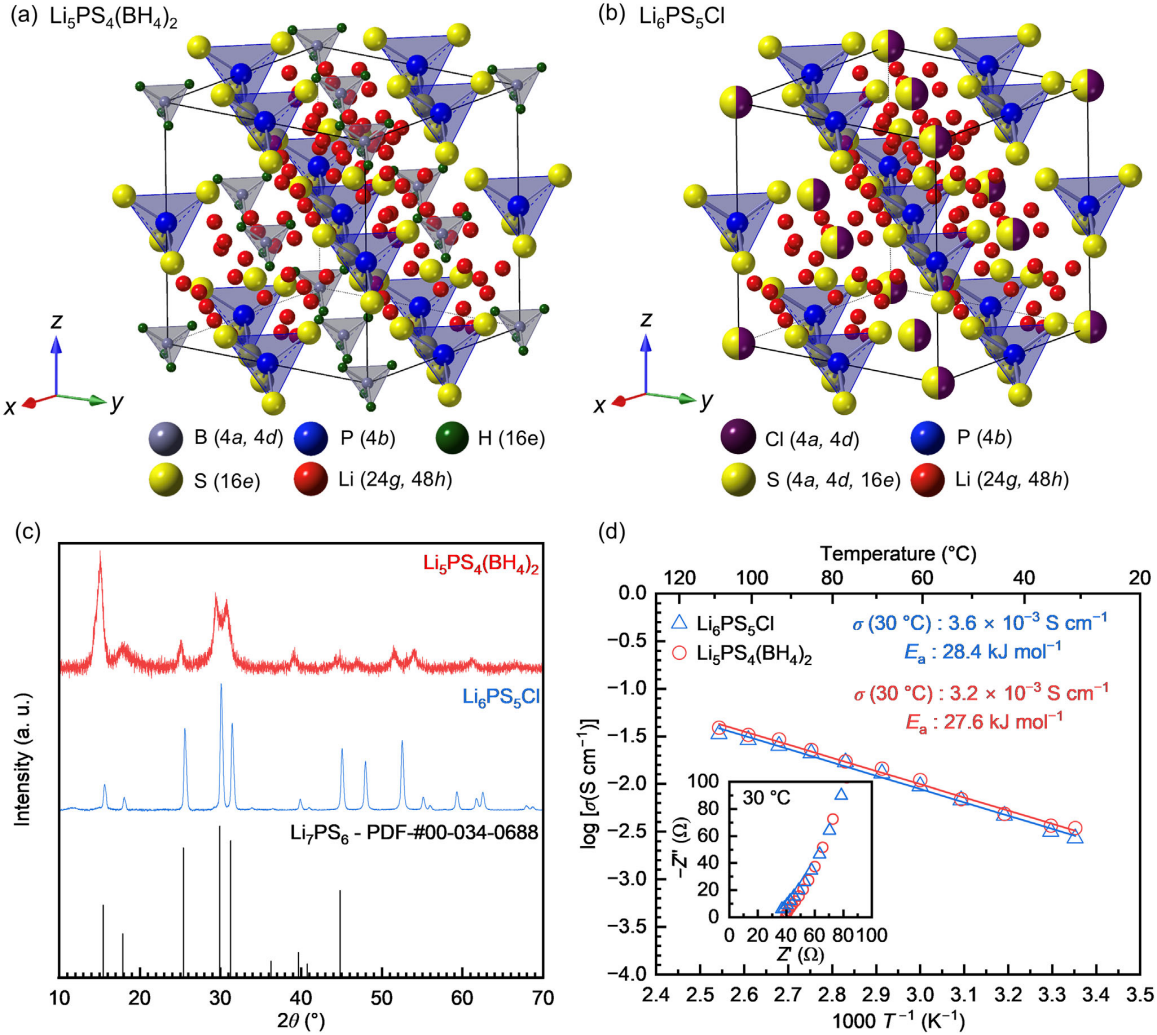
(a) Crystal structure of Li_6_PS_5_Cl (space group *F* $\bar{4}$3*m*). (b) Crystal structure of argyrodite‐type Li_5_PS_4_(BH_4_)_2_ (space group *F* $\bar{4}$3*m*). In both electrolytes, yellow, red, blue, purple, dark indigo, and dark green spheres denote S, Li, P, Cl, B, and H atoms, respectively. (c) XRD patterns of Li_5_PS_4_(BH_4_)_2_ and Li_6_PS_5_Cl. (d) Arrhenius plots of Li_5_PS_4_(BH_4_)_2_ and Li_6_PS_5_Cl. The inset in (d) shows the Nyquist plot at 30°C.

Figure 1a,b shows the crystal‐structures of Li_5_PS_4_(BH_4_)_2_ and Li_6_PS_5_Cl. Both compositions adopt a Face‐Centered Cubic (FCC) structure (space group *F* $\bar{4}$3*m*) [33], sharing the same overall framework, with the primary difference arising from the anion substitution. In Li_6_PS_5_Cl, Cl^−^/ S^2−^ anions (Wyckoff 4*a*) form the FCC framework, and the tetrahedral interstitial sites associated with Wyckoff 4*d* are likewise occupied by Cl^−^/S^2−^ anions [34], whereas in Li_5_PS_4_(BH_4_)_2_, both the framework (Wyckoff 4*a*) and the Wyckoff 4*d*‐associated interstitial sites are occupied by BH_4_^−^ [28].

Substitution of the anion at Wyckoff 4*a* and 4*d* sites with BH_4_^−^ introduces a larger polyatomic anion that slightly expands the framework while preserving the argyrodite structure [35]. In both structures, PS_4_^3−^ (P at the Wyckoff 4*b* and S at the Wyckoff 16*e*) form polyhedral [36]. Li ions are mainly distributed over the Wyckoff 48*h* sites in a disordered manner and occupy nearby 24*g* sites during ion migration [37]. As a result, Li ions form a disordered, cage‐like arrangements around the anions at the Wyckoff 4*d* sites [38].

Figure 1c shows the powder XRD patterns of the synthesized solid electrolytes. Li_6_PS_5_Cl shows the characteristic argyrodite reflections, with no residual precursors or impurity phases after synthesis [37]. Li_5_PS_4_(BH_4_)_2_ also displays diffraction peaks corresponding to those of a typical argyrodite structure, indicating BH_4_^−^ incorporation at framework anion sites (Wyckoff 4*a* and 4*d*). These diffraction features, including peak positions and relative intensities are consistent with previous studies [39, 40]. Owing to the larger size of BH_4_^−^ (2.03 Å) [41] relative to S^2−^ (1.86 Å) [42], the diffraction peaks shift systematically toward lower 2*θ* upon BH_4_^−^ substitution.

To further verify BH_4_^−^ incorporation into the argyrodite framework, we performed solid‐state nuclear magnetic resonance (NMR) spectroscopy. In the ^7^Li spectrum (Figure S1), Li_5_PS_4_(BH_4_)_2_ showed a resonance clearly shifted from those of Li_3_PS_4_ and LiBH_4_, indicating a distinct Li environment, consistent with previous reports [29, 43]. In addition, the ^11^B spectrum exhibited a signal distinct from that of LiBH_4_ (Figure S2). Peak fitting indicated that the BH_4_^−^ species occupy the 4*a* and 4*d* sites of the argyrodite structure [29, 43], with a minor LiBH_4_ contribution. Together, these results support that BH_4_^−^ is predominantly incorporated into the argyrodite structure, although a small amount of LiBH_4_ appears to remain.

The particle morphologies of the synthesized electrolytes were examined by field emission scanning electron microscopy (FE‐SEM) (Figures S3 and S4). Li_6_PS_5_Cl consists of irregularly shaped and relatively angular particles, whereas Li_5_PS_4_(BH_4_)_2_ exhibits more rounded and smoother particles. This morphology difference is consistent with a more deformable nature of the hydride material, which facilitates intimate contact among particles [44, 45].

The ionic conductivity of Li_6_PS_5_Cl and Li_5_PS_4_(BH_4_)_2_ were evaluated over a temperature range of 25–120°C via electrochemical impedance spectroscopy (EIS). As shown in the Arrhenius plot (Figure 1d), the ionic conductivity at 30°C is 3.6 × 10^−3^ S cm^−1^ for Li_6_PS_5_Cl and 3.2 × 10^−3^ S cm^−1^ for Li_5_PS_4_(BH_4_)_2_. Both electrolytes exhibit linear temperature dependence without discontinuities, consistent with stable superionic conduction. The activation energies were estimated to be 28.4 and 27.6 kJ mol^−1^ for Li_6_PS_5_Cl and Li_5_PS_4_(BH_4_)_2_, respectively. These relatively low activation energies indicate facile Li ion diffusion within both electrolyte frameworks, confirming that BH_4_^−^ substitution maintains the structural integrity and high ionic conductivity characteristic of argyrodite‐type sulfide electrolytes.

### 2.2 Chemical/Electrochemical Characterization at Li/Solid Electrolyte Interface

**FIGURE 2:**
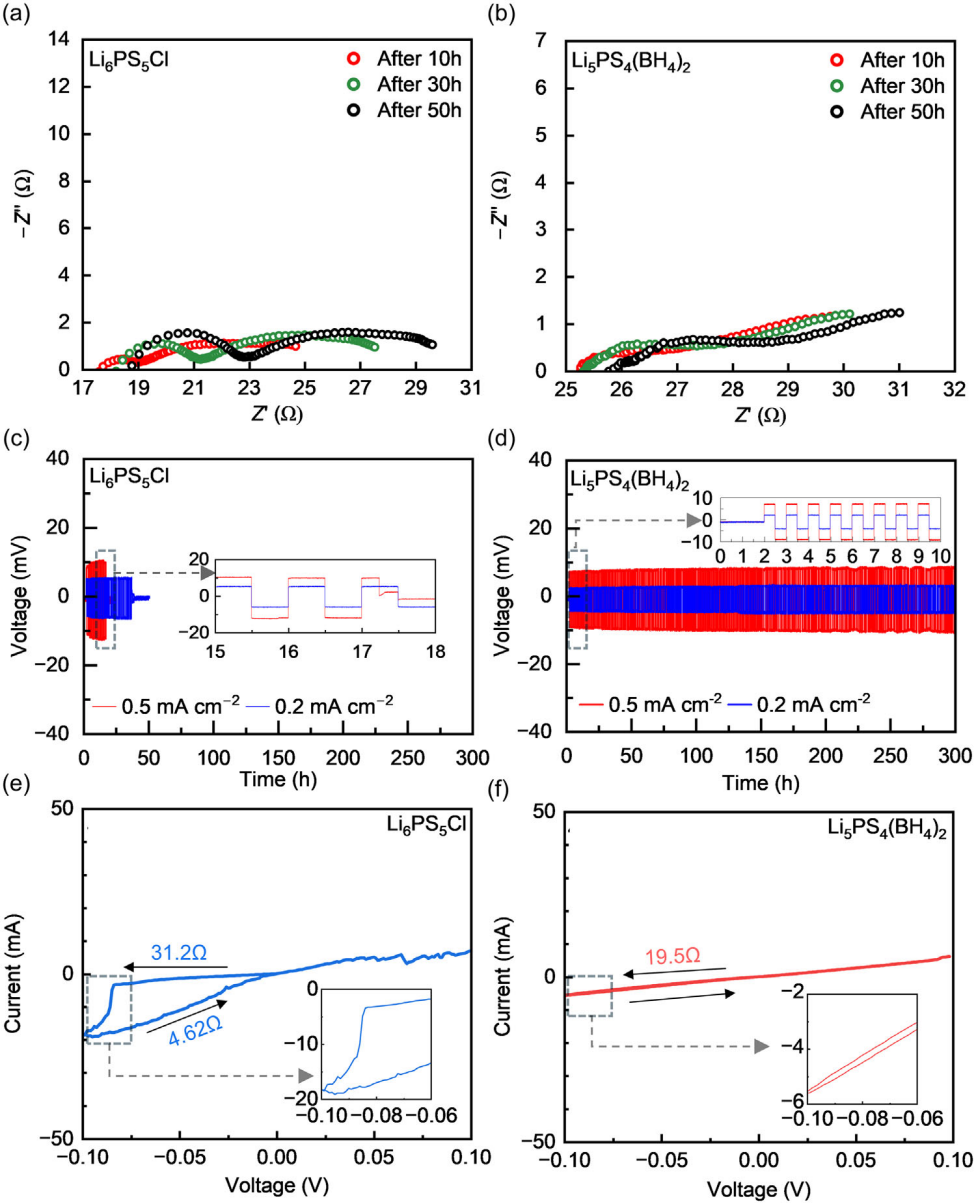
Time‐dependent EIS of Li‐symmetric cells using (a) Li_6_PS_5_Cl and (b) Li_5_PS_4_(BH_4_)_2_ solid electrolytes over 50 h. The red, green, and black plots correspond to measurements after 10, 30, and 50 h, respectively. Galvanostatic cycling profiles at current densities of 0.2 and 0.5 mA cm^−2^ for Li‐symmetric cells using (c) Li_6_PS_5_Cl and (d) Li_5_PS_4_(BH_4_)_2_. CV results of Li‐symmetric cells measured at a scan rate of 0.1 mV s^−1^ using (e) Li_6_PS_5_Cl and (f) Li_5_PS_4_(BH_4_)_2_.

Interfacial reactions at the Li/solid electrolyte interface were investigated by EIS, galvanostatic Li plating and stripping, and cyclic voltammetry (CV). First, we used Li‐symmetric cells to probe chemically driven changes at the Li/solid electrolyte interface. Time‐dependent EIS was performed under open‐circuit conditions, without applying any external potential, to quantify the resulting changes in interfacial resistance (Figure 2a,b). The resulting Nyquist plots were fitted using an equivalent circuit model consisting of one resistance (*R*_1_) and two parallel combinations of constant phase elements (CPEs) and resistances (*R*_2_ and *R*_3_) (Figure S5a). *R*_1_ corresponds to the bulk resistance of the solid electrolyte. *R*_2_ and *R*_3_ in the parallel circuits represent interfacial and charge‐transfer resistances, respectively [46].

In the case of the Li_6_PS_5_Cl‐based cell, *R*_1_, *R*_2_, and *R*_3_ gradually increased over time, with a pronounced increase in *R*_2_, as evidenced by the increase in the diameter of the first semicircle in the Nyquist plots (Figure 2a; Figure S5b), indicating progressive interfacial degradation. The Li_5_PS_4_(BH_4_)_2_‐based cell also exhibited a time‐dependent increase in both interfacial and charge‐transfer resistances, showing a similar overall trend to Li_6_PS_5_Cl (Figure 2b; Figure S6). Taken together, these results indicate that chemically driven interfacial reactions occur in both electrolytes upon contact with Li metal.

Next, to probe electrochemically driven interfacial behavior, we performed Li plating/stripping tests under galvanostatic conditions. The cells were cycled for 300 h at current densities of 0.2 and 0.5 mA cm^−2^ to evaluate whether Li ion transport across the Li/solid electrolyte interphase remains efficient under extended operation. As shown in Figure 2c, the Li_6_PS_5_Cl‐based cell exhibited relatively large initial overpotentials across all current densities. At 0.5 mA cm^−2^, the overpotential increased during the first few cycles and then gradually decreased, but the cell ultimately short‐circuited around 17 h. Even at a low current density of 0.2 mA cm^−2^, Li plating/stripping remained unstable.

In contrast, the symmetric cell based on Li_5_PS_4_(BH_4_)_2_ demonstrated significantly more stable behavior (Figure 2d). At 0.2 mA cm^−2^, the overpotential remained constant over 300 h. Notably, under a higher current density of 0.5 mA cm^−2^, the overpotential maintained a consistent level over the 300‐h test. The critical current density (CCD) profiles of Li_6_PS_5_Cl and Li_5_PS_4_(BH_4_)_2_ (Figure S7) highlight their different electrochemical stability. For Li_6_PS_5_Cl, an abrupt voltage drop at 2.0 mA cm^−2^ indicates short‐circuiting during the stepwise current test. In contrast, Li_5_PS_4_(BH_4_)_2_ maintains stable cycling up to 3.1 mA cm^−2^, demonstrating a higher CCD, comparable to previously reported values for hydride‐substituted argyrodites [43]. These electrochemical results indicate that Li_5_PS_4_(BH_4_)_2_ exhibits enhanced interfacial stability under electrochemical operation.

Subsequently, we performed cyclic voltammetry (CV) at a scan rate of 0.1 mV s^−1^ within a narrow potential window (−100 to 100 mV) to sensitively probe electrochemical reactions at the interface under continuous potential variation [47]. As shown in Figure 2e, the CV profile of Li_6_PS_5_Cl exhibits a gradual increase in current when cycling to a lower voltage (−100 mV), followed by a sharp current rise near −0.09 V, a behavior consistent with previous report [47]. The calculated cell resistance from the inverse slope was estimated to be 31.2 Ω. However, the reverse scan revealed a sudden drop in resistance to 4.62 Ω, suggesting the occurrence of a micro‐short in the cell. These results suggest the evolution of interfacial processes that accelerate dendrite growth during electrochemical cycling [47]. Meanwhile, Li_5_PS_4_(BH_4_)_2_ displayed a nearly linear and symmetric CV response across the entire voltage range (Figure 2f), with no abrupt current changes or redox peaks. This CV profile suggests that the interface formed during the initial electrochemical reaction remains stable without additional signatures of side reactions.

Overall, these chemical and electrochemical measurements clarify how the two electrolytes differ in their interfacial behavior with Li metal. Time‐dependent EIS under open‐circuit conditions shows that both Li_6_PS_5_Cl and Li_5_PS_4_(BH_4_)_2_ undergo continuous chemically driven interfacial degradation upon contact with Li metal. However, under electrochemical operation, their behavior diverges. For Li_6_PS_5_Cl, the sharp current rise observed in the CV indicates that coupled chemical and electrochemical side reactions drive persistent electrolyte decomposition at the interface. In contrast, Li_5_PS_4_(BH_4_)_2_ maintains relatively stable CV responses and Li^+^ transport during electrochemical cycling, indicating a less reactive and more electrochemically robust interface. These results suggest that BH_4_^−^ substitution does not eliminate chemical reactivity toward Li metal but instead leads to a marked difference in interfacial reactivity under electrochemical conditions.

### 2.3 Electrochemical Interphase Formation at the Li/Solid Electrolyte Interface

For the design of all‐solid‐state Li metal batteries, a stable Li/solid electrolyte interface is critical, and extensive efforts have been devoted to achieving it [19, 48]. Because interfacial behavior is influenced by multiple factors, including both chemical and electrochemical reactions, it is important to understand how the interface evolves. In the previous section, we showed that two electrolytes, Li_6_PS_5_Cl and Li_5_PS_4_(BH_4_)_2_, exhibit distinct chemical and electrochemical interfacial responses. Building on these results, we next examine how these reactions are associated with the structures changes of the Li/solid electrolyte interface.

**FIGURE 3:**
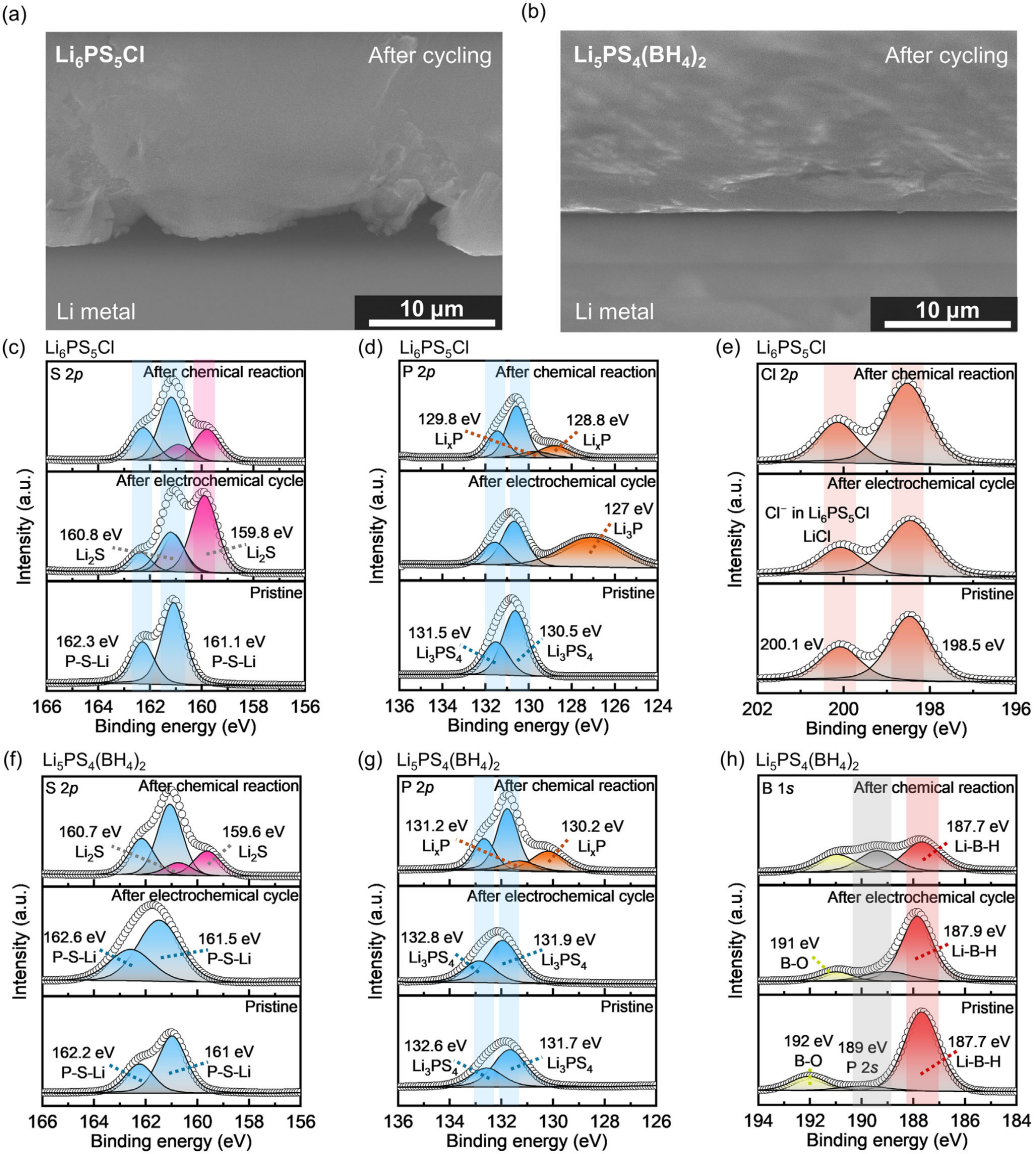
Cross‐sectional SEM images of the Li/electrolyte interface after one CV cycle for (a) Li_6_PS_5_Cl and (b) Li_5_PS_4_(BH_4_)_2_ (scale bar: 10 µm). (c−e) S 2*p*, P 2*p*, and Cl 2*p* XPS spectra of the Li/Li_6_PS_5_Cl interface; (f−h) S 2*p*, P 2*p*, and B 1*s* XPS spectra of the Li/Li_5_PS_4_(BH_4_)_2_ interface. Each set of spectra was collected from the pellet surface before Li contact (bottom), from the Li/electrolyte interface after one CV cycle (middle), and from the Li/electrolyte interface after 100 h of physical contact (top).

We first examined the Li/solid electrolyte interface before (Figure S8) and after cycling using cross‐sectional SEM measurements. As shown in Figure 3a, after electrochemical operation, the Li_6_PS_5_Cl‐based symmetric cell exhibits an unstable interphase characterized by heterogeneous interfacial contact, a non‐planar interface, and locally recessed void‐like features. These morphological observations are consistent with the unstable interfacial behavior identified in the electrochemical measurements.

In contrast, Li_5_PS_4_(BH_4_)_2_‐based symmetric cell maintained a flat, compact, and well‐adhered interface after cycling, without visible delamination or structural disruption (Figure 3b). These results confirm that Li_5_PS_4_(BH_4_)_2_ exhibits superior interfacial continuity and morphological stability, consistent with its stable electrochemical behavior observed across all testing conditions.

To further examine the bonding states at the Li/electrolyte interface beyond the morphological observations, X‐ray photoelectron spectroscopy (XPS) analyses were performed. For each electrolyte, spectra were collected in the pristine state (bottom) and after a single CV cycle (middle), as shown in Figure 3c–h. In order to examine whether similar interfacial changes arise under purely chemical contact without electrochemical operation, we conducted additional XPS measurements after 100 h of contact between Li metal and the solid electrolyte. The results are shown in the top section of Figure 3c–h.

In the pristine Li_6_PS_5_Cl electrolyte, the S 2*p* spectrum shows a doublet at 161.1 and 162.3 eV assigned to P─S─Li bonds (Figure 3c), and the P 2*p* spectrum exhibits a doublet at 130.5 and 131.5 eV corresponding to Li_3_PS_4_ (Figure 3d), confirming the characteristic peaks of the argyrodite Li_6_PS_5_Cl electrolyte [10]. After cycling, the S 2*p* region shows strong growth of the Li_2_S doublet peak near 159.8 eV, evidencing substantial interfacial reduction [49]. In the P 2*p* region, new peaks corresponding to Li_3_P (127 eV) emerge, suggesting reductive cleavage of the P─S framework [50]. Under purely chemical contact conditions with Li metal, Li_2_S‐ and Li_3_P‐related components are also observed in the S 2*p* and P 2*p* spectra, although with lower intensity than in the electrochemically cycled sample. These results indicate that decomposition of Li_6_PS_5_Cl occurs under both chemical and electrochemical conditions, and that electrochemical operation further accelerates side reactions at the interface.

Previous reports have suggested LiCl formation in the Cl 2*p* region during the decomposition of Li_6_PS_5_Cl [10]. However, due to the similar binding energies of LiCl and Cl^−^ in Li_6_PS_5_Cl, clear identification was not possible in this study (Figure 3e). The Li 1*s* spectra also exhibit an additional peak at 54.4 eV, which is assigned to Li_2_S and Li_3_P species formed as side products from the decomposition of Li_3_PS_4_ (Figure S9).

For pristine Li_5_PS_4_(BH_4_)_2_ electrolyte, the S 2*p* spectrum shows a doublet at 161 and 162.2 eV assigned to P─S─Li bonds (Figure 3f). The P 2*p* spectrum exhibits a doublet at 131.7 and 132.6 eV corresponding to Li_3_PS_4_ species (Figure 3g), similar to those observed in Li_6_PS_5_Cl, confirming that the PS_4_^3−^ framework is preserved upon hydride substitution. After cycling, the S 2*p* region does not show distinct Li_2_S‐related peaks, and the P 2*p* region does not exhibit clear signals attributable to Li*_x_* P or Li_3_P. These observations indicate that parasitic decomposition reactions at the Li_5_PS_4_(BH_4_)_2_ interface are effectively suppressed.

Notably, Li_5_PS_4_(BH_4_)_2_ exhibits different behavior under purely chemical contact. When Li metal is contacted without electrochemical operation, the S 2*p* and P 2*p* spectra display Li_2_S‐ and Li*_x_* P‐related components that are not detected after electrochemical cycling. Li 1*s* spectra also show additional signals assigned to reduced species, further confirming chemically driven interfacial decomposition (Figure S10). These results suggests that BH_4_^−^ substitution does not inherently eliminate the tendency for chemical decomposition. However, once an electrochemical bias is applied, simple chemical reduction no longer dominates the interphase evolution, and the interface rapidly evolves into a different interphase state that suppresses further decomposition.

To further understand the contribution of BH_4_^−^ species to the interphase, B 1*s* XPS measurements were performed (Figure 3h). In the pristine state, a prominent peak near 187.7 eV is consistently assigned to Li─B─H species in Li_5_PS_4_(BH_4_)_2_, while a weaker feature around 189 eV can be attributed to the P 2*s* signal. Additionally, weaker signals around 191–192 eV arise from boron oxides formed either by brief air exposure or by moisture‐induced hydrolysis during transfer [32].

After electrochemical cycling, the B 1*s* spectrum shows noticeable changes. The Li─B─H peak shifts slightly to higher binding energy from 187.7 to 187.9 eV, suggesting its enhanced bonding under electrochemical bias. Importantly, this Li–B–H feature remains strong, indicating that BH_4_^−^‐derived species are still dominant near the interface under electrochemical operation.

In addition, the Li 1*s* spectra of the pristine sample reveal a peak near 56.4 eV, which can be assigned to Li─B─H species (Figure S10) [51]. After electrochemical cycling, the Li─B─H peak also shifts to higher binding energy (∼56.6 eV) and increases in intensity. Overall, the observed evolution of the Li─B─H related features in the B 1*s* and Li 1*s* spectra supports the view that, under electrochemical bias, BH_4_^−^ units react with Li to form a BH_4_^−^‐rich interphase at the interface. Furthermore, small shifts toward higher binding energy are also observed in the P 2*p* and S 2*p* regions after electrochemical cycling, indicating that the electrochemically formed interphase modifies the overall electronic environment at the interface.

In contrast, purely chemical contact with Li metal results in a substantially diminished Li─B─H signal, while other species become dominant. The absence of any binding‐energy shifts relative to the pristine Li─B─H peak indicates that this pathway primarily reflects direct degradation rather than the formation of a new interphase structure. Collectively, these observations show that only electrochemical operation produces a modified interphase in which BH_4_^−^‐derived species remain abundant near the interface. This BH_4_^−^‐rich interphase is closely associated with suppressed side reactions and improved preservation of P─S bonding in the argyrodite structure.

**FIGURE 4:**
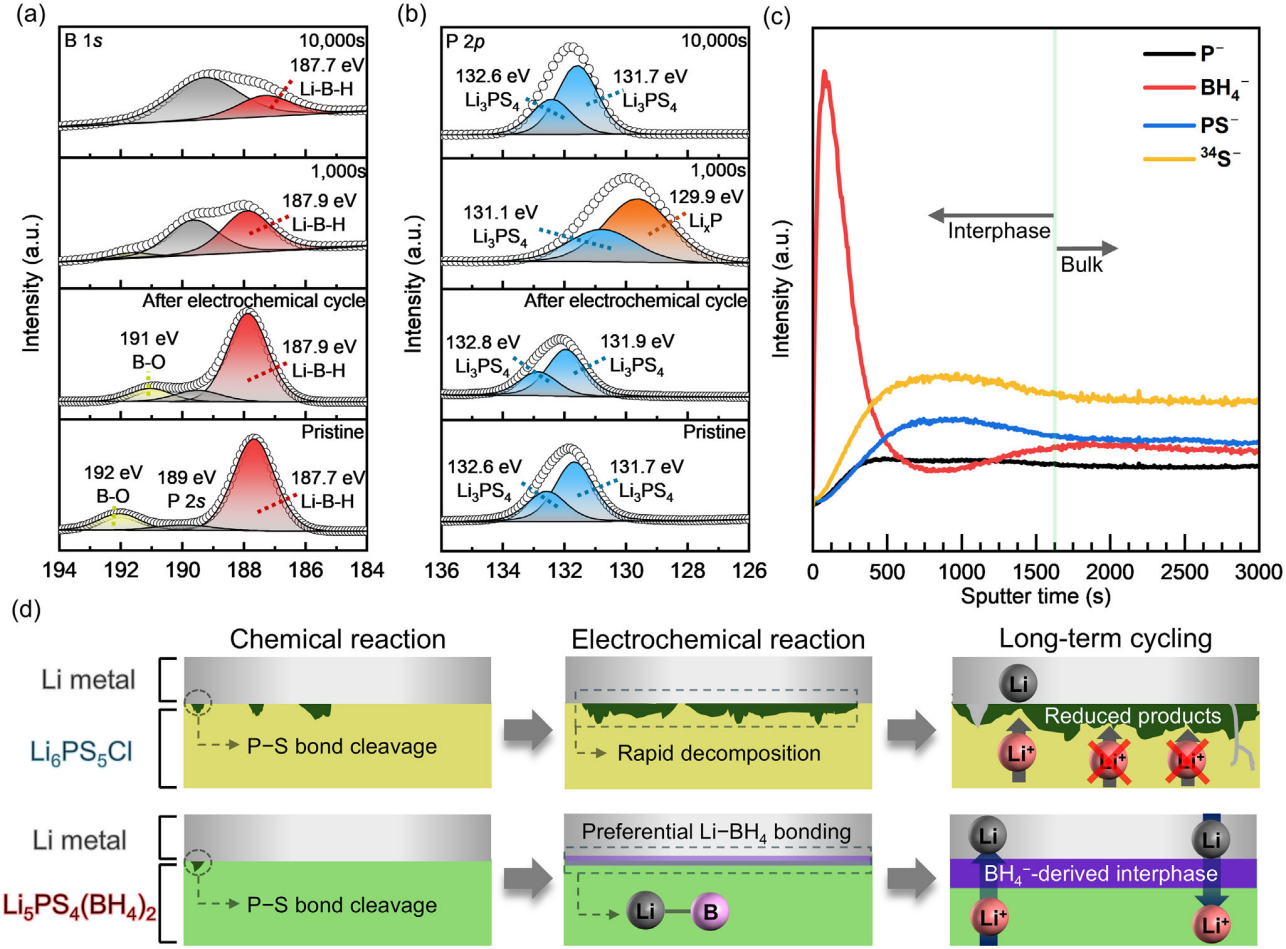
XPS depth‐profiling spectra of Li/Li_5_PS_4_(BH_4_)_2_ interfaces after CV. (a) B 1*s* and (b) P 2*p* spectra of Li_5_PS_4_(BH_4_)_2_, respectively. For each core level, the spectra are plotted from bottom to top as follows: pristine Li_5_PS_4_(BH_4_)_2_ pellet surface, Li/Li_5_PS_4_(BH_4_)_2_ interface after CV without sputtering, and the same interface after 2 kV Ar^+^ sputtering for 1000 and 10 000 s. Increasing sputtering time indicates deeper regions from the interface toward the bulk. (c) ToF‐SIMS depth profile of the electrochemically formed Li/Li_5_PS_4_(BH_4_)_2_ interphase, showing the depth‐dependent evolution of selected fragment ions. The black, red, blue, and yellow curves correspond to P^−^, BH_4_^−^, PS^−^, and ^34^S^−^, respectively. (d) Schematic illustration of interfacial evolution at the Li/Li_6_PS_5_Cl and Li/Li_5_PS_4_(BH_4_)_2_ interfaces.

To further understand the reaction pathways within this interface, XPS depth profiling was performed on the Li_5_PS_4_(BH_4_)_2_ electrolyte after CV (Figure 4a,b). From bottom to top, the four XPS spectra correspond to the pristine electrolyte, the interface after electrochemical cycling, and the electrochemically cycled samples after Ar^+^ etching for 1000 and 10 000 s, respectively. As the etching time increased, the probed region moved gradually from the near‐surface interfacial region toward the bulk of the electrolyte.

The B 1*s* depth profiles reveal that the Li─B─H component is most pronounced at the immediate Li‐contacted interface and gradually decreases with longer Ar^+^ etching times, indicating that the BH_4_^−^‐derived interphase is mainly formed near the interface (after 1000s etching) rather than extending into the bulk of the electrolyte. Near the interface (after 1000s etching), the Li─B─H peak also exhibits a small shift toward higher binding energy. For longer Ar^+^ etching times (after 10 000s etching), the Li─B─H intensity remains lower, while the binding energy converges toward that of pristine Li_5_PS_4_(BH_4_)_2_. These depth‐dependent trends indicate that, away from the interface, the remaining Li–B–H species experience a bulk‐like chemical environment, even though BH_4_^−^‐derived species are partially depleted in this region.

In the P 2*p* region, a Li*_x_* P signal is not observed at the outermost surface but appears only at an intermediate depth (after 1000s etching), reflecting partial P─S bond cleavage during the initial chemical reaction with Li metal. However, this signal disappears upon deeper etching (after 10 000s etching), and the PS_4_^3−^‐related peaks again dominate in the bulk region. This trend suggests that, although the chemical reaction involves multiple anion species (S^2−^, PS_4_^3−^, and BH_4_^−^), subsequent electrochemical operation shifts the interfacial pathway toward preferential BH_4_^−^‐derived reactions, effectively protecting the P─S framework from further decomposition.

To complement the XPS depth‐profiling results and directly visualize the spatial distribution of the interfacial species, time‐of‐flight secondary‐ion mass spectrometry (ToF‐SIMS) depth profiling was performed on the electrochemically formed interphase (Figure 4c). The interphase was generated by contacting Li metal on one side of the SSE pellet and an inert current collector on the opposite side, followed by CV down to −0.1 V. The 3D renderings show a distinguishable spatial separation between the interphase region and the deeper bulk‐like region (Figure S11).

As shown in Figure 4c, the dominant BH_4_^−^ signal in the initial sputtering stage indicates the presence of a borohydride‐derived, Li─B─H‐rich interphase in the outermost region. As sputtering proceeds, the BH_4_^−^ signal gradually decreases, whereas the PS^−^ signal increases and reaches a plateau at around 1600 s. This trend suggests that, after passing through the BH_4_^−^‐rich interphase, the probed region approaches a more bulk‐like PS_4_^3−^‐related environment. In addition, the decrease in the BH_4_^−^ signal and the relative increase in the S^−^ and P^−^ signals after approximately 400 s suggest the presence of sulfur‐ and phosphorus‐containing interfacial reduction products. This interpretation is consistent with the XPS depth‐profiling results, in which the Li─B─H signal decreases upon etching while Li*_x_* P‐like species appear at intermediate depth.

Taken together, these results suggest that the electrochemically formed interphase is compositionally graded, consisting of an outer Li─B─H‐rich region and an underlying layer in which sulfur/phosphorus‐based reduction products coexist. These depth‐dependent changes further suggest that the outer interphase likely plays an important role in sustaining stable long‐term electrochemical cycling. This interpretation is consistent with previous reports that electrochemically formed stable interphases support prolonged cycling stability [52, 53, 54, 55]. A schematic summary of the interfacial evolution is provided in Figure 4d, highlighting the chemical and electrochemical interphase formation at the Li/solid electrolyte interface.

### 2.4 All‐Solid‐State Li Metal Battery

**FIGURE 5:**
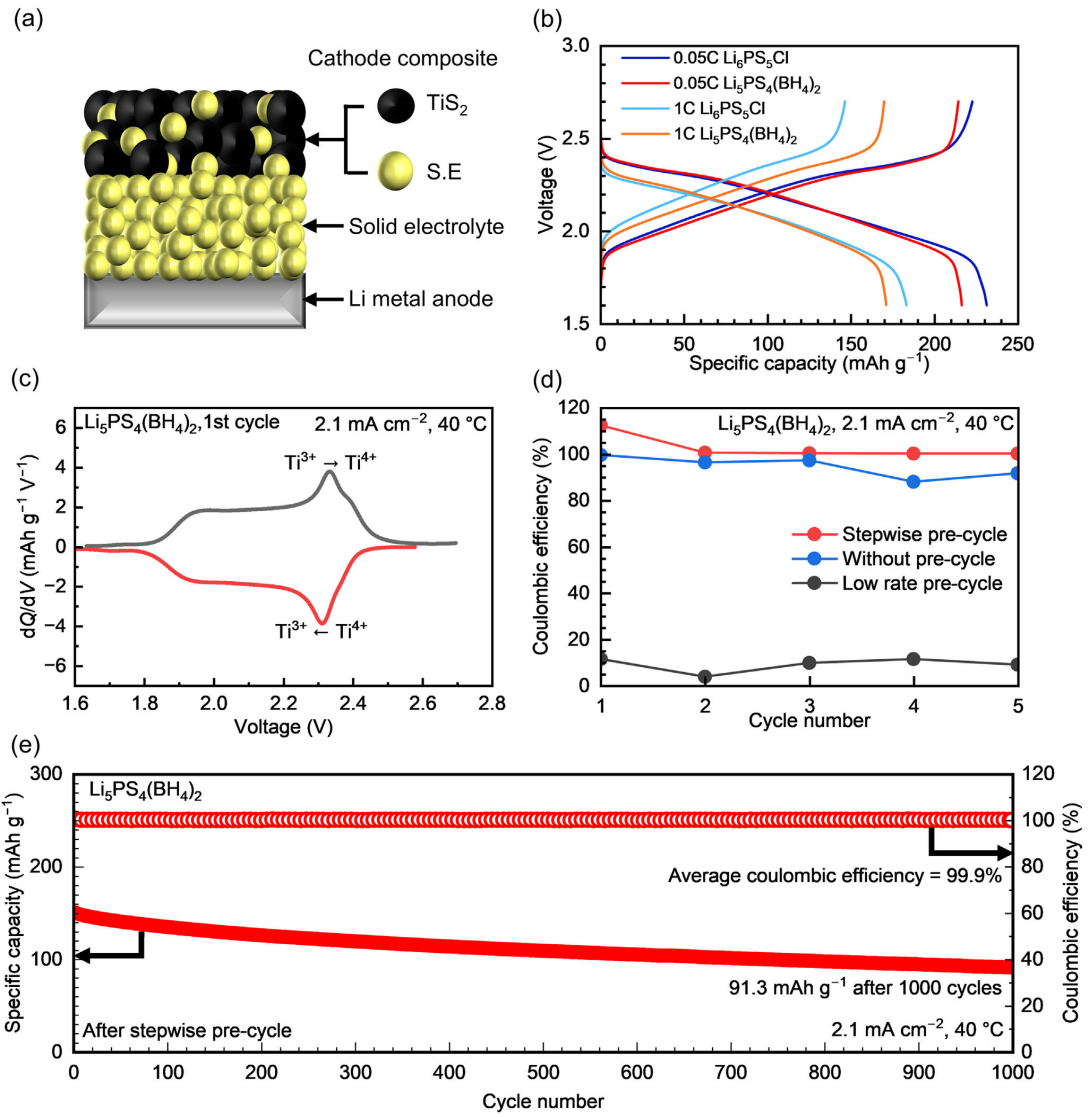
(a) Schematic illustration of the TiS_2_/Li all‐solid‐state battery. (b) First charge–discharge profiles at 0.05C and 1C for cells using Li_5_PS_4_(BH_4_)_2_ and Li_6_PS_5_Cl. (c) d*Q*/d*V* curve of the first charge–discharge cycle at 1C for the Li_5_PS_4_(BH_4_)_2_‐based cell. (d) Coulombic efficiency of Li_5_PS_4_(BH_4_)_2_ full cells during the initial cycles at 1C (current density 2.1 mA cm^−2^) after different pre‐cycling protocols: stepwise pre‐cycle (charging and discharging at 0.1C, 0.2C, 0.5C, and again 0.1C before switching to 1C, red), without pre‐cycle (blue), and low‐rate pre‐cycle (four cycles at 0.1C before switching to 1C, black). (e) Cycling performance of discharge capacity and Coulombic efficiency at 1C for the Li_5_PS_4_(BH_4_)_2_‐based cell after stepwise pre‐cycling.

A TiS_2_/Li_5_PS_4_(BH_4_)_2_/Li full cell was assembled to evaluate the suitability of Li_5_PS_4_(BH_4_)_2_ as a solid electrolyte for all‐solid‐state batteries and to further assess its interfacial stability against Li metal (Figure 5a). For quantitative analysis, we also assembled a Li_6_PS_5_Cl‐based cell under the same conditions. Given that borohydride‐type complex hydride electrolytes can exhibit limited oxidative stability at high potentials [56], the choice of cathode is important to separate cathode‐driven effects from the interfacial processes of interest. Although stable high‐voltage cycling has been reported using Li_6_PS_5_Cl‐containing cathode composites with a borohydride‐based electrolyte [25], we selected TiS_2_ in this work to minimize cathode‐induced effects and to focus on Li‐metal interfacial behavior under low‐potential conditions.

Figure 5b shows the first‐cycle voltage profile measured at 0.05C and 1C at 40°C. At 0.05C, the Li_6_PS_5_Cl‐based cell delivered a first discharge capacity of 232 mAh g^−1^, corresponding to approximately 97.1% of the theoretical capacity of TiS_2_ (239 mAh g^−1^), while the Li_5_PS_4_(BH_4_)_2_‐based cell delivered 221 mAh g^−1^, corresponding to 92.5% of the theoretical value. However, at a high rate (1C), the Li_5_PS_4_(BH_4_)_2_‐based cell exhibits a noticeably higher charging capacity than the Li_6_PS_5_Cl‐based cell, indicating improved reversibility and a higher Coulombic efficiency during cycling. In addition, the d*Q*/d*V* plots for the Li_5_PS_4_(BH_4_)_2_‐based cell show clearly resolved redox peaks during cycling (Figure 5c), consistent with this enhanced reversibility. This tendency becomes even more pronounced in long‐term cycling. As shown in Figure S12, the Li_5_PS_4_(BH_4_)_2_‐based cell sustained excellent cycling performance for 200 cycles at 0.5C. By contrast, the Li_6_PS_5_Cl cell operated for only 6 cycles before failing, indicating cycling instability.

Interestingly, even when the same Li_5_PS_4_(BH_4_)_2_ electrolyte was used, the pre‐cycling protocol had a pronounced impact on high‐rate stability. In the stepwise pre‐cycling protocol, the cell was cycled sequentially at 0.1C, 0.2C, and 0.5C and then returned to 0.1C before being operated at 1C. As shown in Figure 5d, when the cell was subsequently driven at 1C, the Coulombic efficiency remained close to 100% during the first few cycles, whereas cells that were driven at 1C without any pre‐cycling, or after pre‐cycles at a low rate (0.1C) only, exhibited markedly lower and more fluctuating Coulombic efficiencies.

Furthermore, after the stepwise pre‐cycle was applied, the Li_5_PS_4_(BH_4_)_2_ full cell also showed stable long‐term cycling at 1C, corresponding to a high current density of 2.1 mA cm^−2^, with the Coulombic efficiency remaining close to 100% over 600 cycles (Figure 5e). This current density is substantially higher than values typically reported for all‐solid‐state Li metal batteries employing BH_4_^−^‐substituted argyrodite electrolytes [25, 27, 57]. Such enhanced high‐rate performance is consistent with the formation of the BH_4_^−^‐derived interphase identified in this study, suggesting that this interphase supports stabilization of the Li/solid electrolyte interface under high‐current operation. By contrast, cells without this stepwise protocol displayed unstable long‐term cycling under the same high‐current conditions (Figures S13 and S14). Moreover, Li_5_PS_4_(BH_4_)_2_‐based symmetric cell likewise maintained stable cycling for 100 h at 2 mA cm^−2^ after stepwise pre‐cycling, consistent with the full‐cell results (Figure S15).

These results suggest that the formation of the BH_4_^−^‐derived interphase is sensitive to how the applied current is increased. When the current is raised stepwise, BH_4_^−^ species can preferentially react with Li without excessive overpotential, forming a relatively uniform BH_4_^−^‐derived interphase that enables high Coulombic efficiency and stable cycling even at high current. In contrast, applying a high current immediately without sufficient formation, likely induces local overpotentials and heterogeneous reactions at the interface, so that decomposition of the P─S bonds becomes dominant before a BH_4_^−^‐based interphase is fully established. These findings demonstrate that stable Li metal full cells can be achieved without relying on additional protective coatings or interlayers at the Li metal anode.

Building on these results, we now relate our findings to previous studies on BH_4_^−^‐introduced argyrodite electrolytes. Attempts to introduce BH_4_^−^ into argyrodite‐type sulfide electrolytes have already demonstrated considerable potential in several excellent earlier reports [35, 40, 57]. These studies have elucidated that introducing BH_4_^−^ into argyrodite electrolytes enhances ionic conductivity and improves physical interfacial contact with Li metal. Moreover, BH_4_^−^ doping through mechanical mixing has been reported to form a tri‐layered interphase containing a B‐rich intermediate region, which suppresses electronic leakage while preserving efficient ionic transport [32].

Our study is distinguished by showing that, when BH_4_^−^ is incorporated into the argyrodite lattice rather than simply mixed, the interfacial reaction pathway under electrochemical bias and the resulting interphase structure are markedly different. In other words, the structure and chemistry of the electrochemically formed interphase strongly depend on how BH_4_^−^ is introduced into the argyrodite electrolyte.

Taken together with these previous studies, our results indicate that complex hydride anion substitution, combined with a rationally engineered electrochemical reactions at the Li interface, enables the formation of interphases that can suppress electronic leakage while maintaining efficient Li^+^ transport at the interface. Continued investigation of electrochemically formed interphases involving complex hydride anions is expected to deepen our understanding of Li metal interfaces and further advance research on all‐solid‐state Li metal batteries.

## 3 Conclusion

In this study, we demonstrated that complex hydride anions play a critical role in governing interfacial stability and electrochemical performance at Li/argyrodite solid electrolyte interfaces. Even when chemically driven decomposition occurs at the Li interface, subsequent electrochemical operation causes BH_4_^−^ species to react rapidly with Li and form a BH_4_^−^‐derived interphase. This interphase suppresses parasitic side reactions and enables highly reversible Li plating and stripping at the interface. When the current is increased gradually, this interphase can form and be maintained in a more stable state, thereby enabling sustained high‐current operation and long‐term cycling in Li metal full cells. Taken together with previous studies, our findings provide new insight into the role of complex hydride anion substitution with suitably tuned electrochemical protocols in stabilizing Li metal interfaces and enabling stable high‐current operation in all‐solid‐state Li metal batteries.

## 4 Experimental Section

### 4.1 Solid electrolyte synthesis

Li_5_PS_4_(BH_4_)_2_ solid electrolyte was synthesized via a two‐step ball‐milling process [39]. In the first step of synthesis, Li_2_S (99.98%, Sigma–Aldrich) and P_2_S_5_ (99%, Sigma–Aldrich) were weighed in a stoichiometric ratio and transferred into an 80 mL zirconia jar with ten zirconia balls (10 mm‐diameter). The mixture was ball‐milled using a planetary mill (PM 100, Retsch GmbH) at 400 rpm for 20 h, with cycles consisting of 15 min milling and 5 min rest. In the second step, LiBH_4_ (95%, Acros Organics) was added to the pre‐milled powder in a stoichiometric amount. The resulting mixture was subjected to additional ball milling under the same conditions as the first step.

Li_6_PS_5_Cl solid electrolyte was synthesized through solid‐state heat‐treatment synthesis method. Li_2_S (99.9%, Sigma–Aldrich), P_2_S_5_ (99%, Sigma–Aldrich), and LiCl (99%, Sigma–Aldrich) were weighed in the stoichiometric ratio and charged into a 45 mL zirconia milling jar together with twenty zirconia balls (10 mm‐diameter). After that, planetary ball milling (Pulverisette 6, Fritsch) was carried out at 350 rpm for 2 h. The resulting powder was uniaxially pressed at 50 MPa to form pellets and heat treated under vacuum at 550°C for 6 h. To control the particle size of the solid electrolyte (*D*_50_: 5.0 µm), wet planetary milling was performed using heptane as the milling medium. All these procedures were conducted under an Ar atmosphere.

### 4.2 Characterization

For crystal structure analysis, X‐ray diffraction (XRD; SmartLab, Rigaku) measurements were performed over the 2*θ* range of 10°–70° with a step size of 0.01° using Cu *K_α_* radiation (wavelength *λ* = 1.5406 Å for *K_α_*_1_ and 1.5444 Å for *K_α_*_2_). The powder for the XRD measurements was loaded into a thin‐walled glass capillary (0.5 mm in diameter) under an Ar atmosphere and sealed using paraffin liquid. Solid‐state nuclear magnetic resonance (NMR; JEOL‐400 MHz spectrometer) was performed to confirm lattice substitution. Each spectra were acquired using a single‐pulse experiment with a 4 mm rotor. Morphology and elemental analyses were conducted using a field emission scanning electron microscopy (FE‐SEM; SNE‐4500 M, Sec). Cross‐sectional SEM samples were prepared by sectioning the samples using a microtome (DORCO LIVING VINA Co., Ltd). The exposed cross‐sectional surface were further polished, when necessary, using an Ar‐ion beam milling system (IM4000II, Hitachi). Ion milling was performed at 2 kV under Ar with a swing angle of ±40° at slow speed. X‐ray photoelectron spectroscopy (XPS; NEXSA, Thermo Fisher Scientific) analysis was carried out after the chemical/electrochemical reaction to investigate the chemical changes occurring at the Li/solid electrolyte interface. Additionally, sputter depth profiling was performed using 2 kV Ar^+^ ions for up to 10 000 s. The samples were retrieved from the cells and transferred to the analysis chamber using an air‐tight holder to prevent air exposure. All sample preparations were conducted under an Ar atmosphere. Time of flight secondary ion mass spectrometry (ToF‐SIMS; M6, ION‐TOF) depth profiling of the Li/SSE interface was conducted in negative ion mode. A Bi_1_^+^ primary ion beam was used for analysis (30 keV; raster size: 50 µm × 50 µm), while a Cs^+^ ion beam was used for sputtering (1 keV; raster size: 150 µm × 150 µm).

### 4.3 Ionic Conductivity Measurement

For ionic conductivity measurements, 100 mg of solid electrolyte powder was placed into a 10 mm‐diameter PET mold (Premium Glass Co., Ltd) and uniaxially pressed at 280 MPa. Subsequently, Au powder (99.9%, Mitsuwa Chemicals) was spread on both sides of the pressed solid electrolyte and further uniaxially pressed at 92.45 MPa. Then, the assembled cells were sealed in a stainless‐steel electrochemical cell and fixed under a constant torque of 4.0 N m (12.7 Mpa) using a torque wrench. Ionic conductivities were measured by the AC impedance method over the temperature range of 25–120°C with an applied frequency of 0.1 Hz to 10 MHz using electrochemical impedance spectroscopy (EIS; MTZ‐35, Biologic).

### 4.4 Electrochemical Analysis and Battery Tests

All chemical and electrochemical reactions were examined using a Li/solid electrolyte/Li symmetric cell. To prepare solid electrolyte pellets, 100 mg of solid electrolyte powder was uniaxially pressed into a 10 mm‐diameter PET mold (Premium Glass Co., Ltd) under a pressure of 400 MPa. Subsequently, Li foils (200 µm, Honjo Metal Co.) were placed on both sides of the solid electrolyte pellet with Ni foils (30 µm, MTI Co.) and uniaxially compressed again at 55 MPa. The assembled cells were sealed in stainless‐steel electrochemical cells and fixed under a constant torque of 4.0 N m (12.7 MPa) using a torque wrench. All cell fabrication processes were conducted under an Ar atmosphere. Critical current density test was conducted under galvanostatic conditions with a fixed electrodeposition and dissolution capacity of 0.5 mAh cm^−2^. The protocol started at a current density of 0.1 mA cm^−2^, followed by stepwise increments of 0.1 mA cm^−2^ until short‐circuiting was observed. At each current step, the deposition and stripping duration was adjusted to maintain the constant areal capacity. To evaluate the electrochemical stability of the solid electrolytes, cyclic voltammetry (CV; ZIVE MP1, WonATech Co.) was performed within a voltage range of −100 to 100 mV at a scan rate of 0.1 mV s^−1^. The cell resistances were investigated using EIS (MTZ‐35, Biologic) over a frequency range of 0.1 Hz to 1 MHz. Li stripping and plating tests were conducted at current densities of 0.2 and 0.5 mA cm^−2^ using a battery tester (WBCS3000, WonATech Co.).

Full cell tests were performed with a TiS_2_/solid electrolyte/Li cell. For cathode composite preparation, TiS_2_ (99%, Sigma–Aldrich) and solid electrolyte powder were mixed in a 7:3 weight ratio by using an agate mortar and pestle for 20 min. For cell fabrication, 100 mg of solid electrolyte powder was placed into a 10 mm‐diameter PET mold (Premium Glass Co., Ltd) and then uniaxially pressed at 280 MPa. Subsequently, 10 mg of the cathode composite was uniformly spread on one side of the solid electrolyte pellet and uniaxially pressed at 530 MPa. Li foil (200 µm, Honjo Metal Co.) was placed on the opposite side as an anode. The assembled full cells were sealed in stainless steel electrochemical cells and fixed at a torque of 5.0 N m (15.9 MPa). All fabrication processes were carried out under an Ar atmosphere. The prepared full cells were cycled within a voltage range of 1.6–2.7 V (*vs*. Li^+^/Li) under CCCV mode using a battery tester (WBCS3000, WonATech Co.). The C‐rate was defined based on 1C = 239 mA g^−1^. All electrochemical measurements were performed at 40°C.

## Conflicts of Interest

The authors declare no conflicts of interest.

## Supporting information




**Supporting File**: advs75514‐sup‐0001‐SuppMat.docx.

## Data Availability

The data that support the findings of this study are available from the corresponding author upon reasonable request.
