# Supplementary material for: Electrochemically Induced Interphase by Complex Hydride Anions in Argyrodite Solid Electrolytes for Stable Lithium Metal All‐Solid‐State Batteries
Source: Adv Sci (Weinh). 2026 May 9:e75514. Online ahead of print. doi: 10.1002/advs.75514 (PMC13335586; doi:10.1002/advs.75514)
Supplement: Supplementary file 1 — Supporting File: advs75514‐sup‐0001‐SuppMat.docx. [file ADVS-9999-e75514-s001.docx]

Supplementary Information

Electrochemically induced interphase by complex hydride anions in argyrodite solid electrolytes for stable lithium metal all-solid-state batteries

*Sangho Lee, Hyunseo Park, Ye-Eun Park, Taehyun Kim, Taegyoung Lee, Taeseung Kim, Seoung Jae Kang, Yerim Chae, Seunghee Joo, Jinkwang Hwang, Kazuhiko Matsumoto, Kyungsu Kim, Woosuk Cho***, and Sangryun Kim**

S.H. Lee, Y. Park, T.H. Kim, T. Lee, T.S. Kim, S. Kang, Y. Chae, S. Joo, S. Kim

Department of Chemistry, Gwangju Institute of Science and Technology (GIST), 123 Cheomdangwagi-ro, Buk-gu, Gwangju, 61005, Republic of Korea

E-mail: sangryun@gist.ac.kr

H. Park, K. Kim, W. Cho

Advanced Batteries Research Center, Korea Electronics Technology Institute (KETI), 25 Saenari-ro, Seongnam 13509, Republic of Korea

J. Hwang, K. Matsumoto

Graduate School of Energy Science, Kyoto University, Yoshida-honmachi, Sakyo-ku, Kyoto 606-8501, Japan

S. Kim

Graduate School of Energy Convergence, Gwangju Institute of Science and Technology (GIST), 123 Cheomdangwagi-ro, Buk-gu, Gwangju, 61005, Republic of Korea.

*Corresponding authors. E-mail: sangryun@gist.ac.kr, [cho4153@keti.re.kr](mailto:cho4153@keti.re.kr)

**
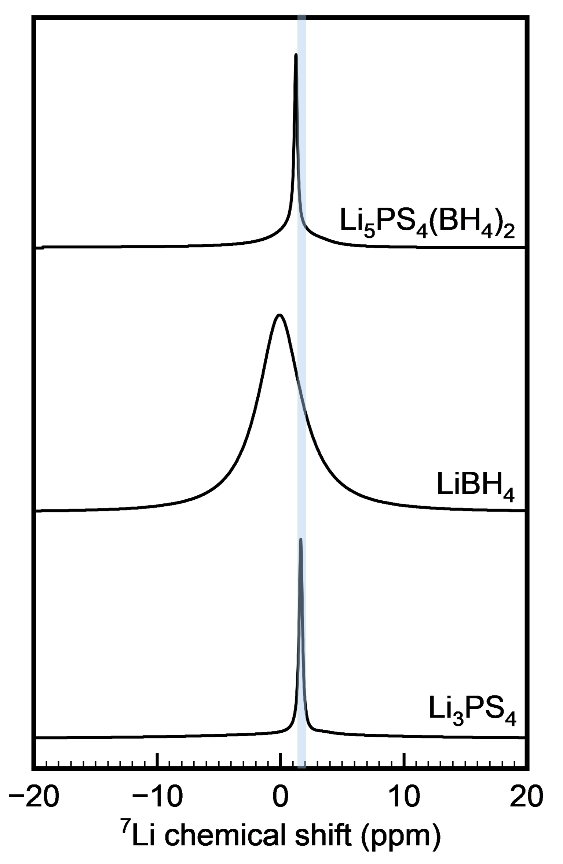
Figure S1.** ^7^Li MAS NMR spectra of Li_3_PS_4_, LiBH_4_, and the synthesized Li_5_PS_4_(BH_4_)_2_.

**
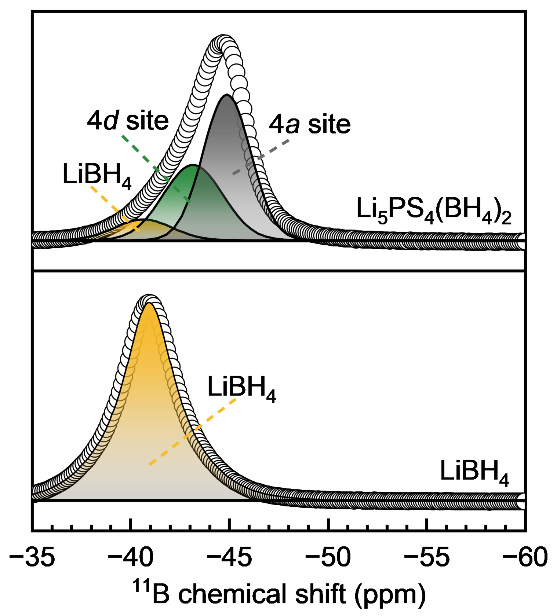
Figure S2.** ^11^B MAS NMR spectra of LiBH_4_ and synthesized Li_5_PS_4_(BH_4_)_2_. The orange region corresponds to LiBH_4_, whereas the green and dark gray regions are assigned to BH_4_^−^ at the 4*d* and 4*a* sites of Li_5_PS_4_(BH_4_)_2_, respectively.


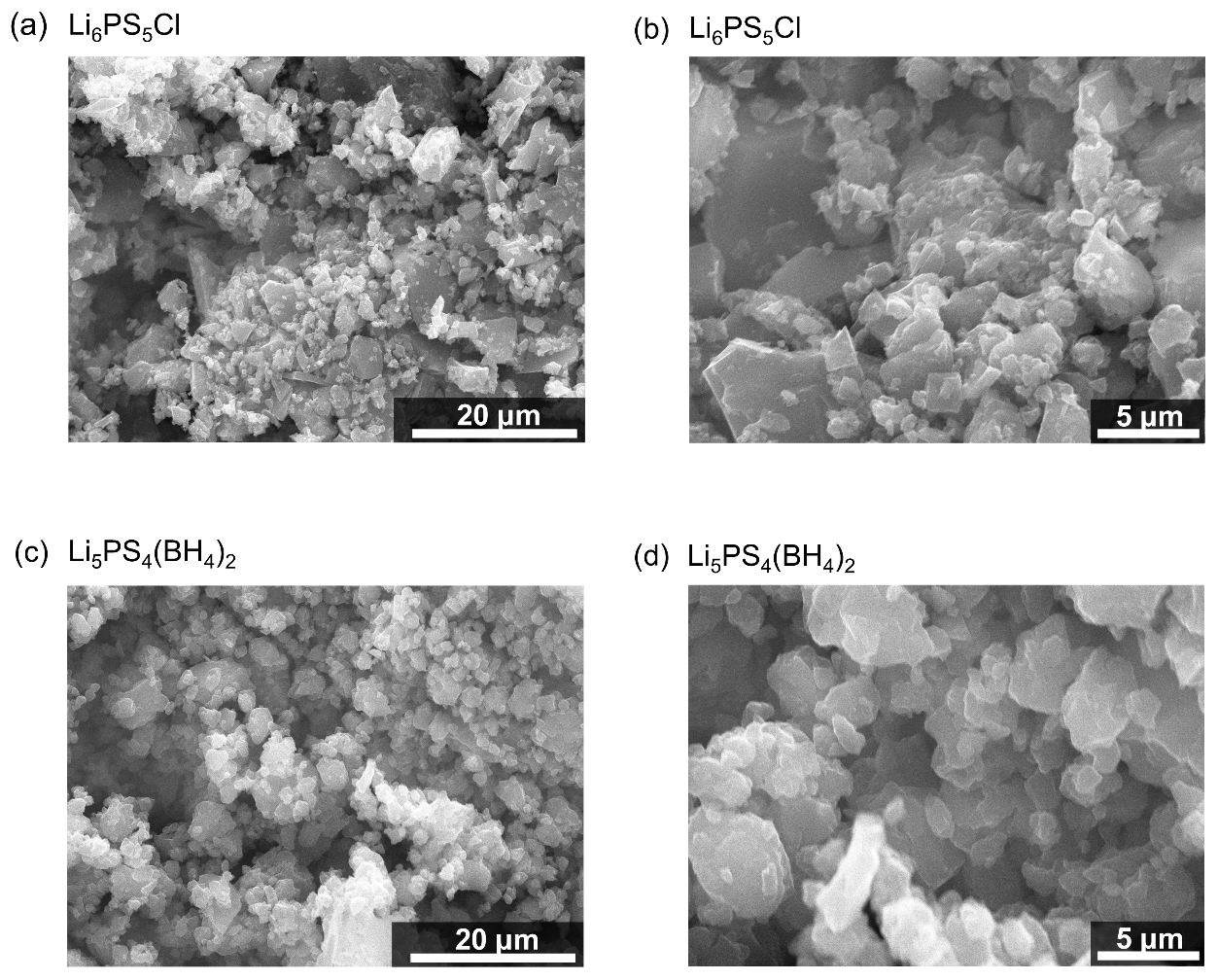


**Figure S3.** FE-SEM images of the argyrodite solid electrolytes. (a) Li_6_PS_5_Cl, scale bar: 20 µm; (b) Li_6_PS_5_Cl, scale bar: 5 µm; (c) Li_5_PS_4_(BH_4_)_2_, scale bar: 20 µm; (d) Li_5_PS_4_(BH_4_)_2_, scale bar: 5 µm.


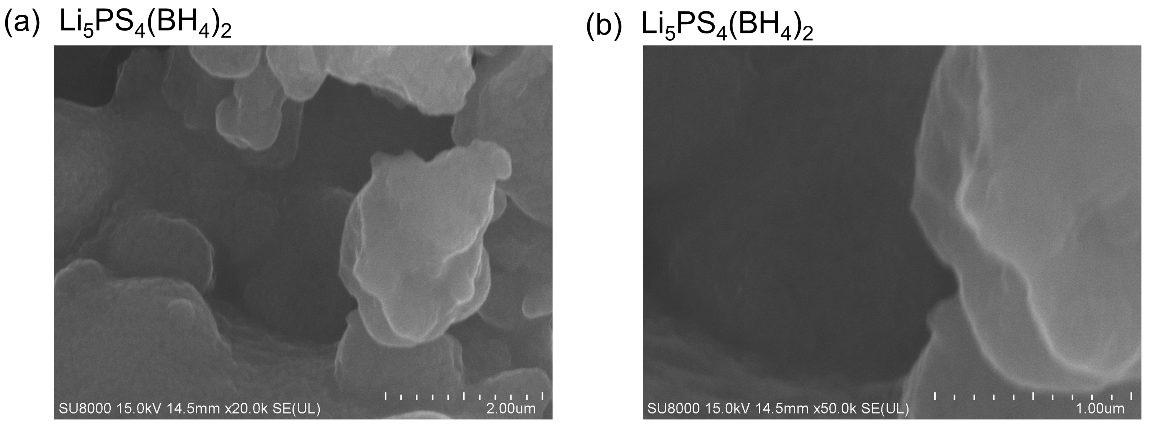


**Figure S4.** High resolution FE-SEM images of the Li_5_PS_4_(BH_4_)_2_. (a) scale bar 2 µm; (b) scale bar 1 µm.


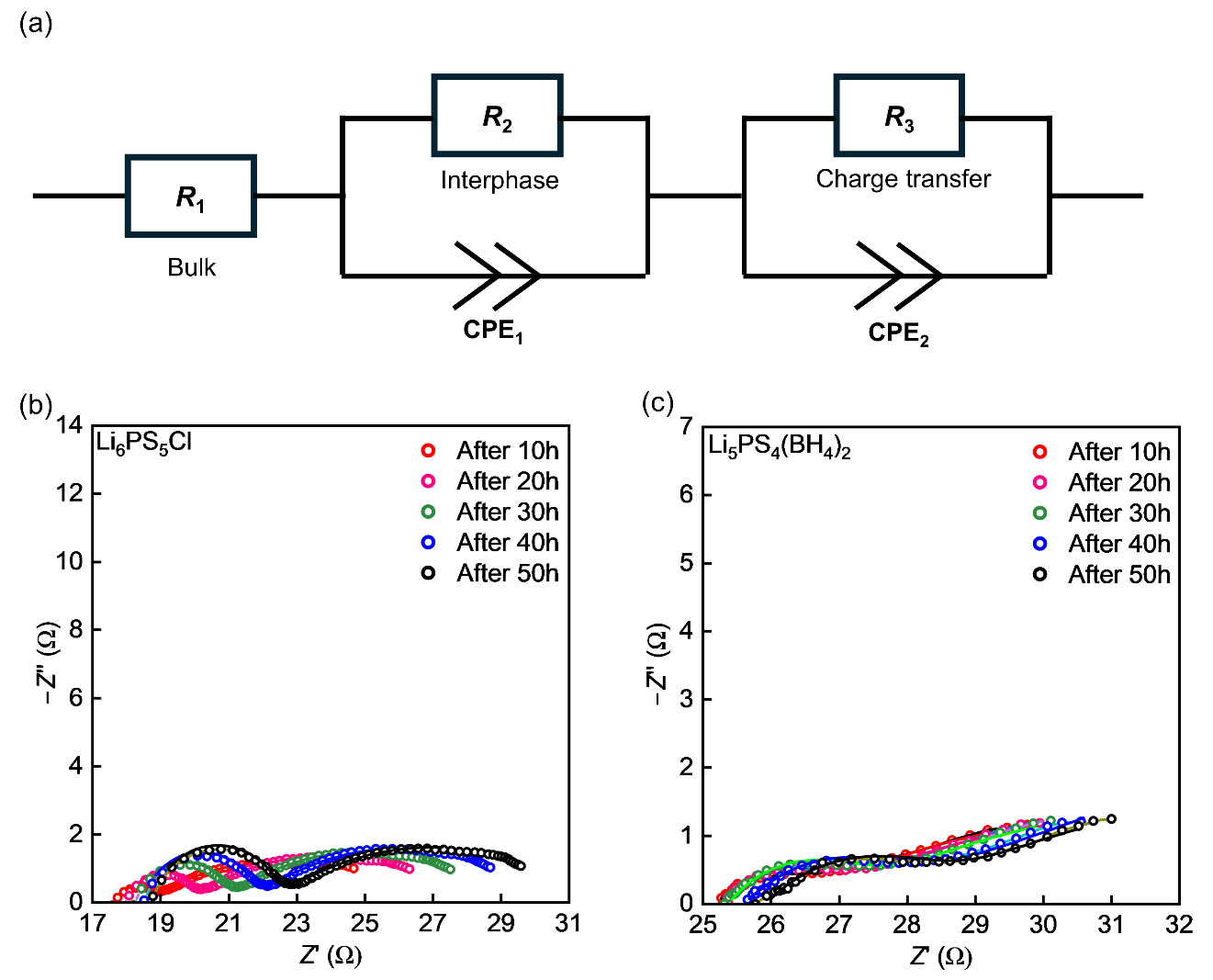


**Figure S5.** (a) The equivalent circuit model used to fit the EIS data. Time-dependent electrochemical impedance spectra of Li-symmetric cells using (b) Li_6_PS_5_Cl and (c) Li_5_PS_4_(BH_4_)_2_ solid electrolytes over 50 hours. The red, pink, green, blue, and black curves correspond to spectra collected after 10, 20, 30, 40, and 50 hours, respectively.


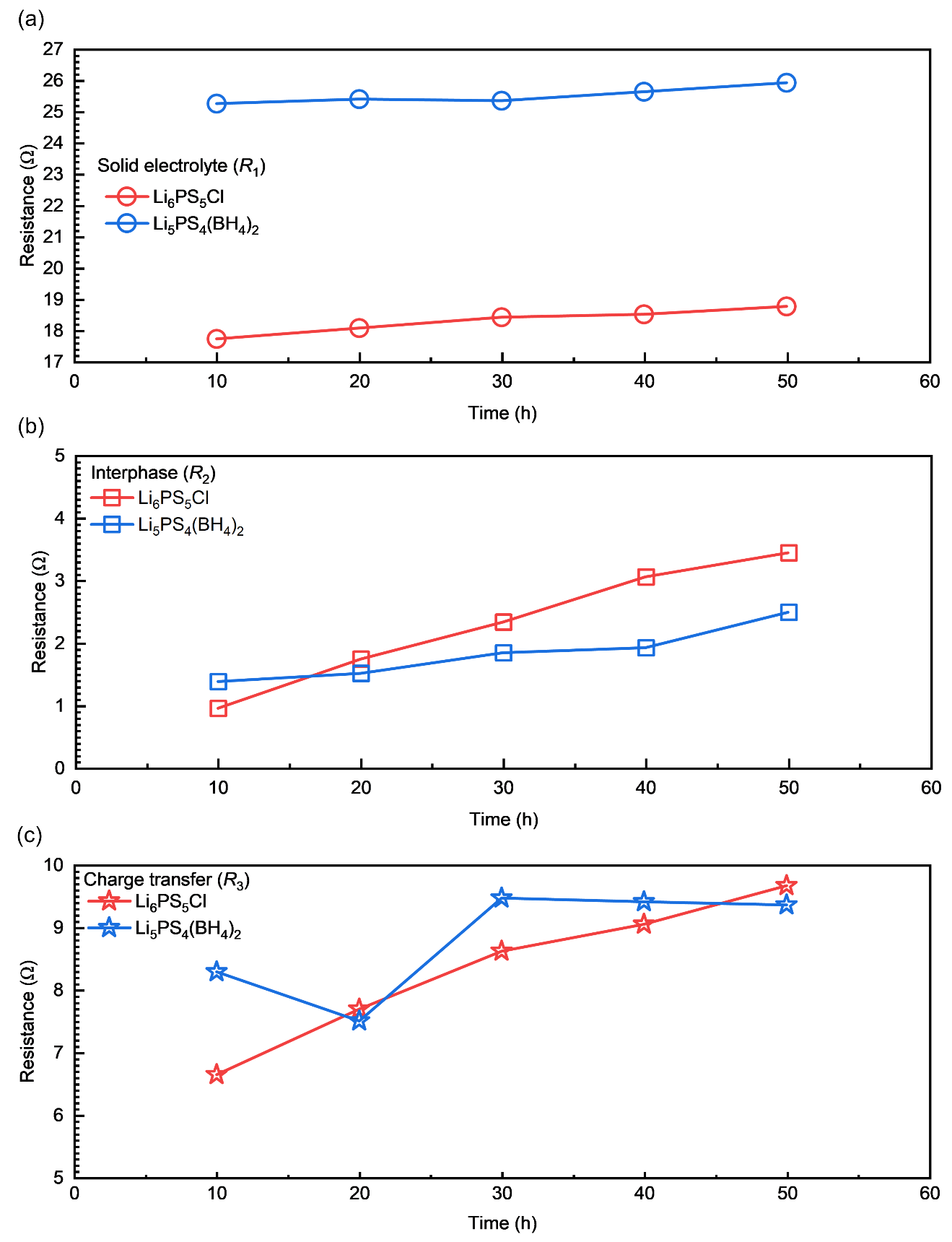


**Figure S6.** Time-dependent EIS fitting parameters for Li_6_PS_5_Cl and Li_5_PS_4_(BH_4_)_2_: (a) solid electrolyte resistance, (b) interphase resistance, and (c) charge-transfer resistance.

**
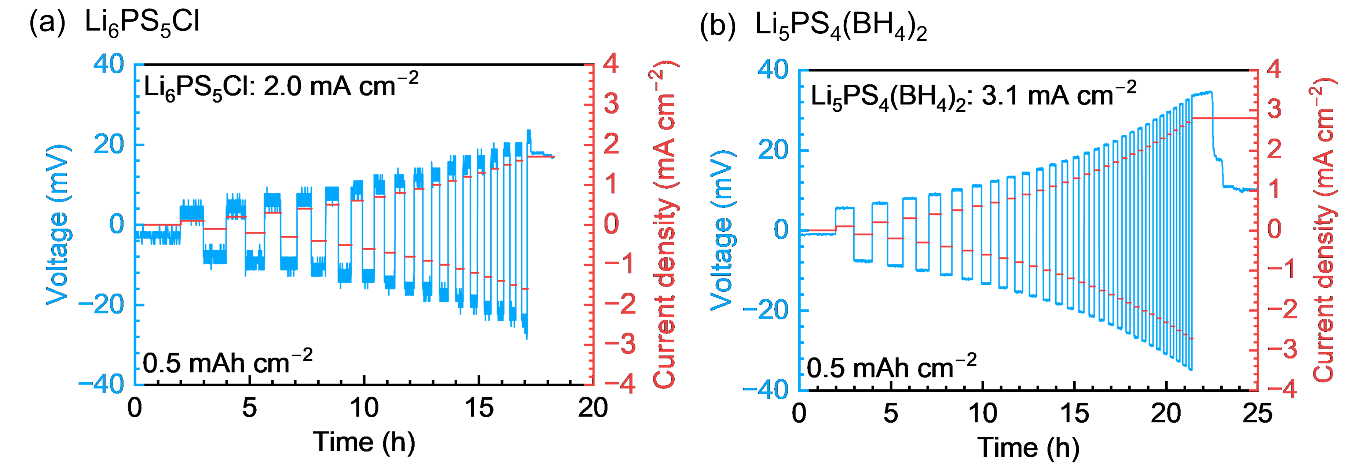
Figure S7.** Critical current density (CCD) measurements of Li/Li symmetric cells using (a) Li_6_PS_5_Cl and (b) Li_5_PS_4_(BH_4_)_2_ solid electrolytes. Stepwise galvanostatic plating/stripping tests were performed with increasing current density until short circuiting occurred.


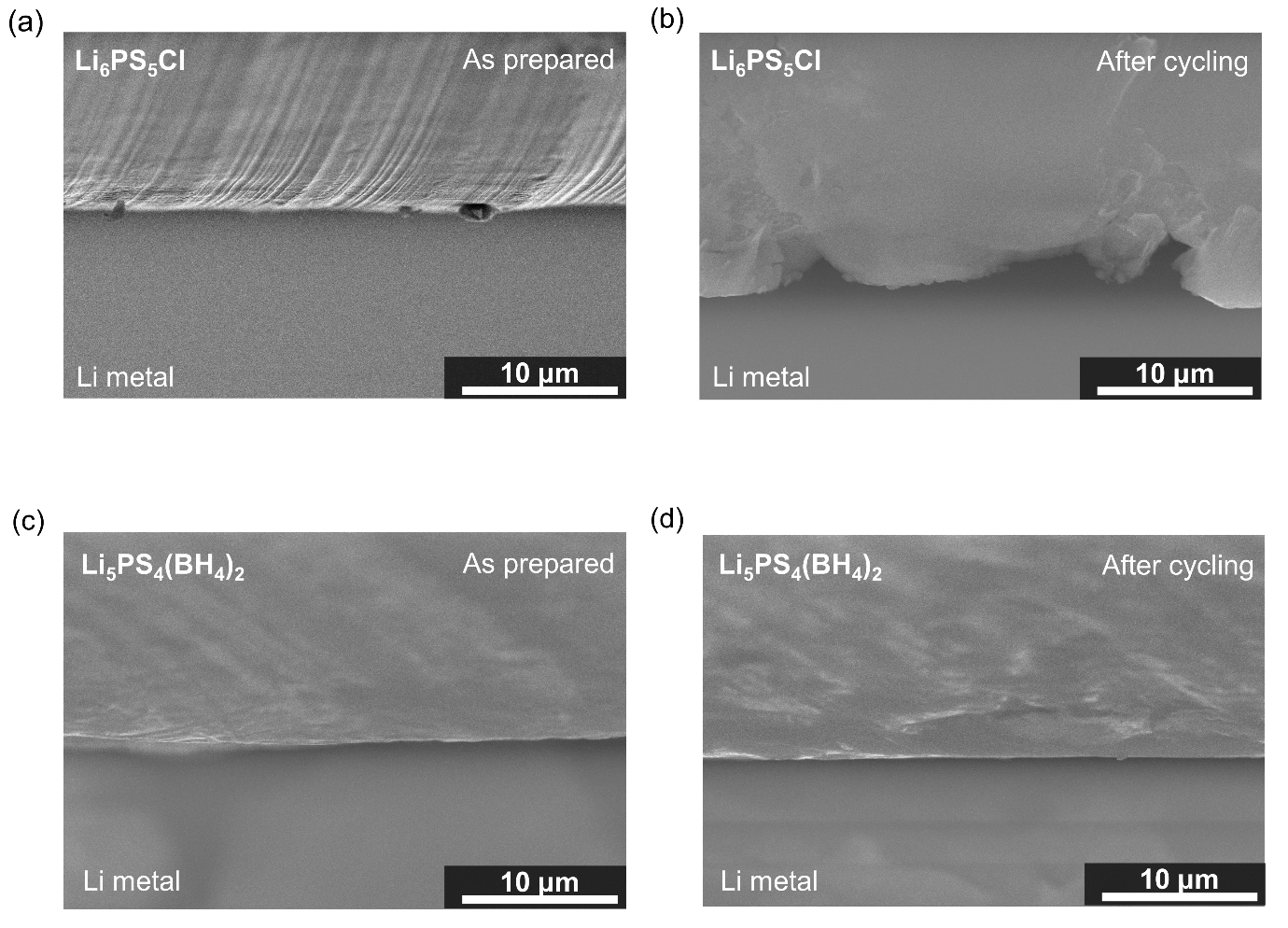
**Figure S8.** Cross-sectional SEM images of the Li/Li_6_PS_5_Cl interface (a) before and (b) after one CV cycle, and the Li/Li_5_PS_4_(BH_4_)_2_ interface (c) before and (d) after one CV cycle (scale bar: 10 μm).


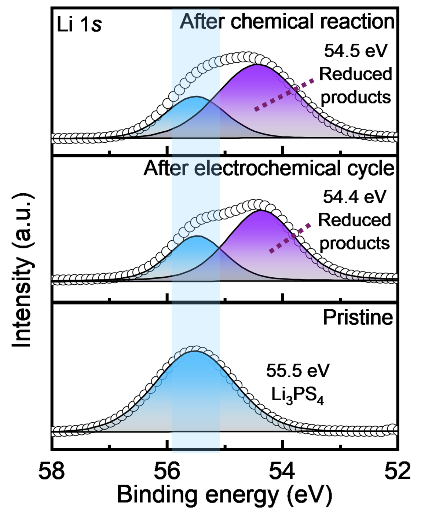


**Figure S9.** XPS spectra of Li 1*s* at the Li/Li_6_PS_5_Cl interface. Each set of spectra was collected from the pellet surface before Li contact (bottom), from the Li/electrolyte interface after one CV cycle (middle), and after 100 h of physical contact (top).

**
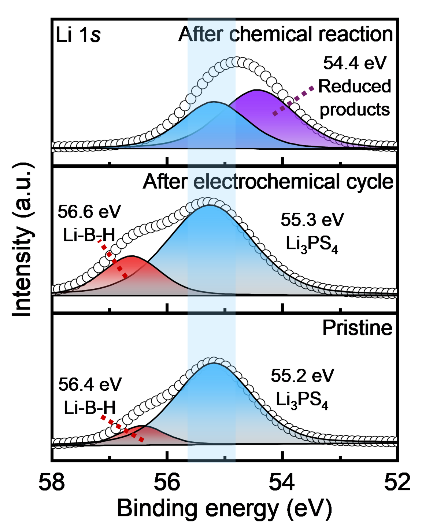
Figure S10.** XPS spectra of Li 1*s* at the Li/Li_5_PS_4_(BH_4_)_2_ interface. Each set of spectra was collected from the pellet surface before Li contact (bottom), from the Li/electrolyte interface after one CV cycle (middle), and after 100 h of physical contact (top).

**
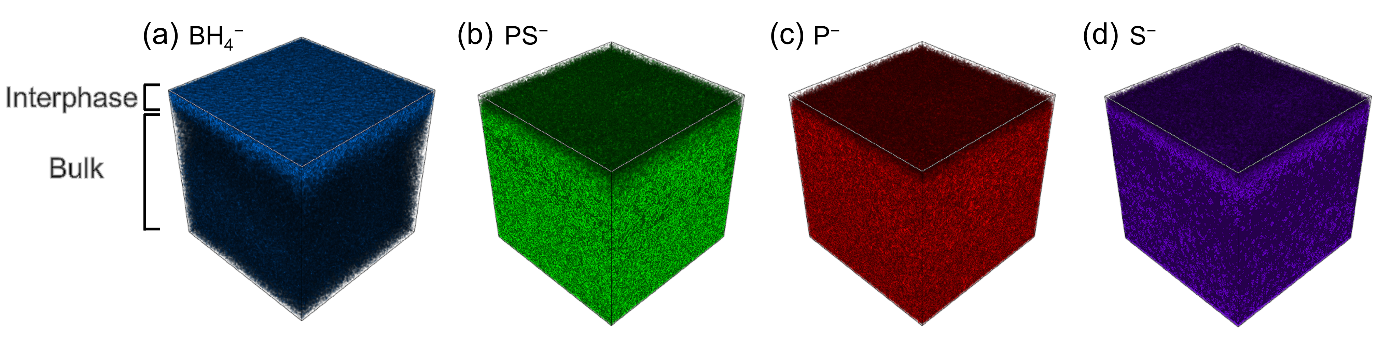
Figure S11.** 3D renderings of the ToF-SIMS depth profile for the electrochemically formed Li/Li_5_PS_4_(BH_4_)_2_ interface: (a) BH_4_^−^, (b) PS^−^, (c) P^−^, and (d) S^−^.

**
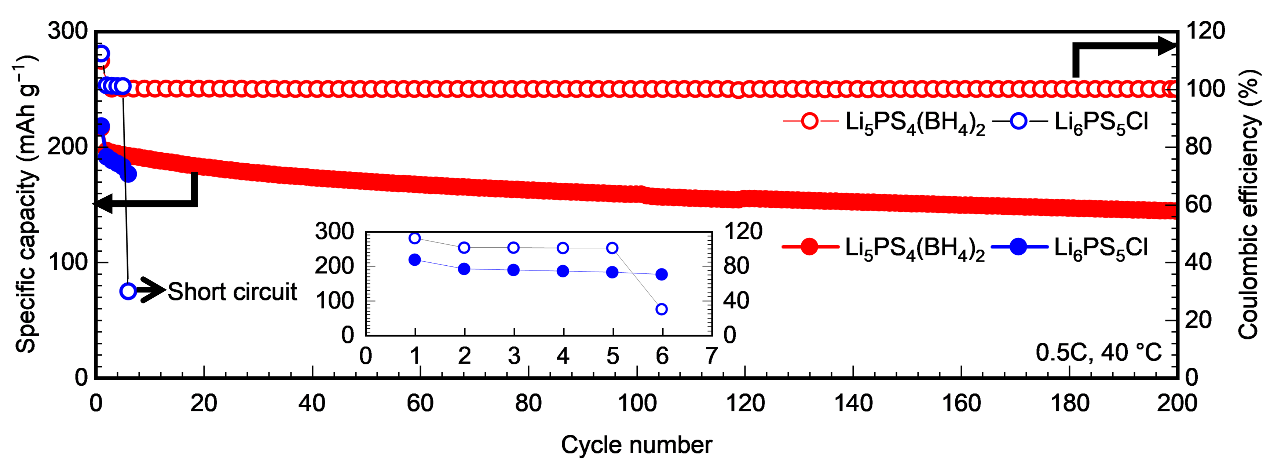
Figure S12.** Cycling performance of discharge capacity and Coulombic efficiency at 0.5C. The inset shows the cyclability of Li_6_PS_5_Cl, with an internal short circuit observed on cycle 6.

**
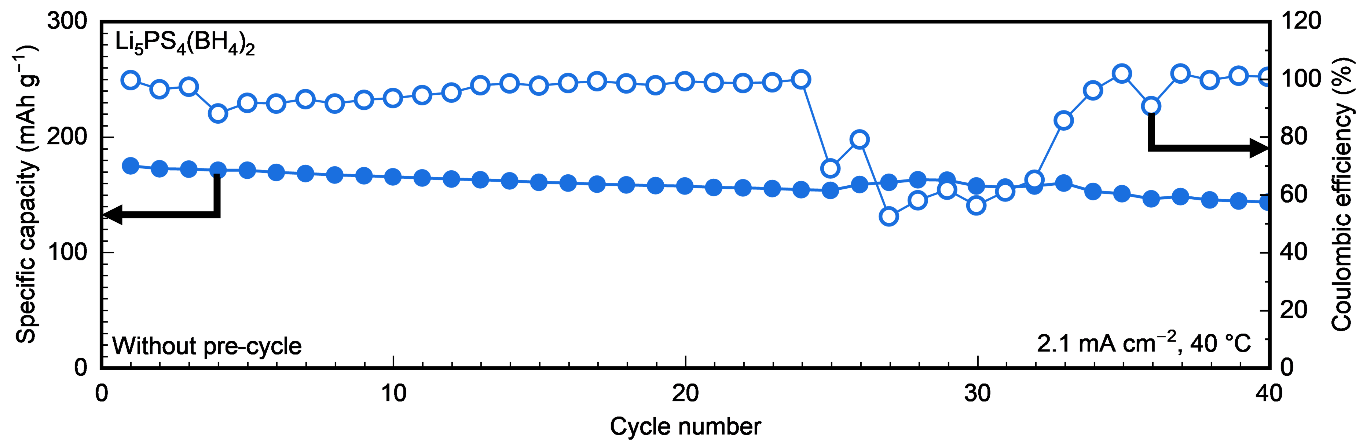
Figure S13.** Cycling performance of discharge capacity and Coulombic efficiency at 1C for the Li_5_PS_4_(BH_4_)_2_-based cell without pre-cycles.

**Figure S14.** Cycling performance of discharge capacity and Coulombic efficiency at 1C for the Li_5_PS_4_(BH_4_)_2_-based cell after four pre-cycles at 0.1C.
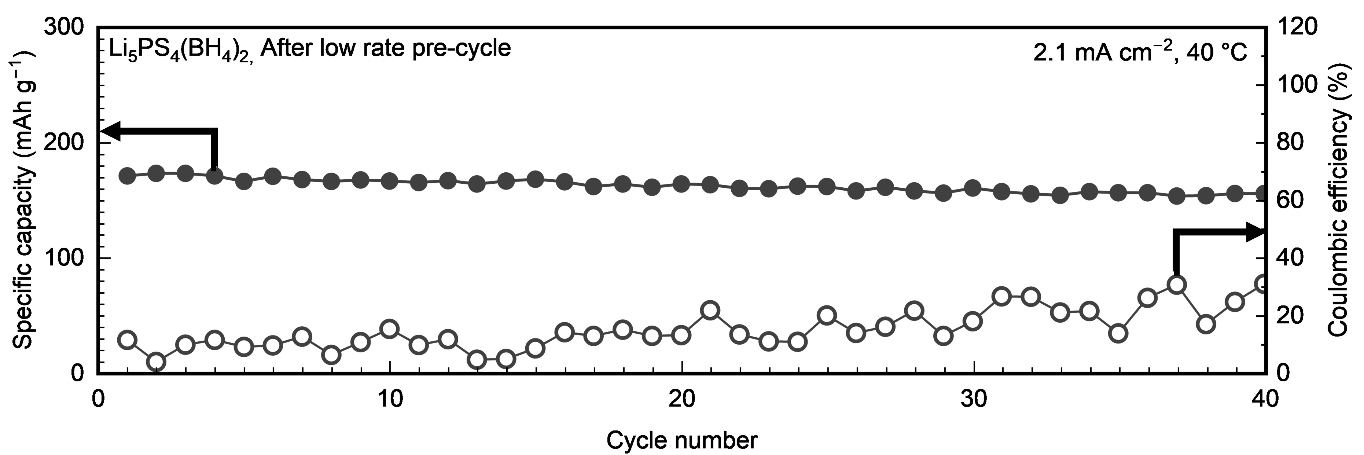


**
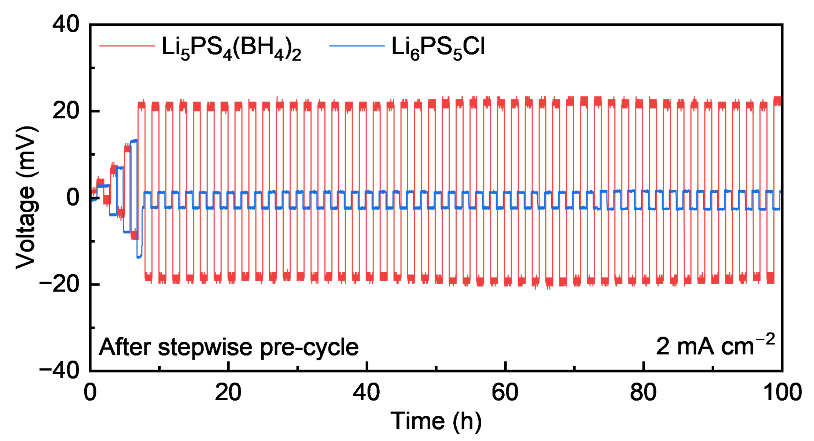
Figure S15.** Galvanostatic cycling profiles of Li-symmetric cells using Li_5_PS_4_(BH_4_)_2_ and Li_6_PS_5_Cl at current densities of 2 mA cm^−2^ after stepwise pre-cycling at 0.2, 0.5, and 1 mA cm^−2^
